# Stress sources and symptoms: the role of gender in a Brazilian university medical school

**DOI:** 10.3389/fpubh.2024.1492229

**Published:** 2025-01-17

**Authors:** Marina Romeo, Antonio Luiz Marques, Montserrat Yepes-Baldó, Sefa Boria-Reverter

**Affiliations:** ^1^Department of Social Psychology and Quantitative Psychology, Research Group on Social, Environmental and Organizactional Psychology PsicoSAO, Universitat de Barcelona, Barcelona, Spain; ^2^Department of Administrative Sciences, Federal University of Minas Gerais, Belo Horizonte, Brazil

**Keywords:** occupational stress, faculty, gender, Brazil, occupational health

## Abstract

**Introduction:**

The main objective of this research was to identify the sources and symptoms of occupational stress among Brazilian university professors and examine the impact of gender on this relationship.

**Methods:**

A total of 81 university professors from a Brazilian Federal University Medical School answered a questionnaire that collected demographic and occupational data, lifestyle information, health issues, and stress symptoms originating from work and personal factors. Univariate statistics, Spearman correlation and the forgotten effects theory were used to analyze the data.

**Results:**

Participants experienced medium-high stress levels from individual factors and low stress levels from work-related factors. Common stress symptoms included fatigue and nervousness. There was a significant correlation between stress sources from work and individual factors, with different symptoms linked to each source. Gender moderated the relationship between work-related stress sources and symptoms and the forgotten effects theory revealed overlooked but significant cause-and-effect relationships.

**Discussion:**

Universities should implement supportive measures and policies that consider the unique challenges faced by faculty, particularly related to gender. These initiatives can create a healthier and more productive work environment for professors and benefit both faculty members and students alike.

## 1 Introduction

In the work context, occupational stress is defined as an individual's reaction to the threats experienced in their work environment (1). These threats, known as stressors, lead to an imbalance between the individual's psychological structure and their occupational environment, affecting their wellbeing (2).

Occupational stress has been considered a major health risk factor for workers worldwide, and more recently for teachers, as well. In this sense, different authors have indicated that occupational stress causes a general deterioration in health status by generating conditions such as gastritis, gastrointestinal diseases (3), hypertension (4), depression (5), and heart disease (6). For organizations, stress can cause work accidents, relationship problems, excessive delays and absences, sick leave, and high turnover, leading the economic costs of stress at work to reach hundreds of billions of dollars a year in many parts of the world (7).

In the last 20 years, research on academic and general staff of universities shows that the phenomenon of occupational stress in universities is alarmingly widespread as a consequence of the increasing pressure to attract external funds, “publish or perish” effect (8), excessive workload (9), poor working conditions (8), low recognition, low wages (10), poor interpersonal relationships and difficulties to deal with student behavior (11).

In the last 3 years, teachers have been subjected to additional sources of stress such as teaching online as a result of COVID. During the lockdown, they had to quickly and profoundly change their teaching methods and how they provided instructional materials to students (12). Contrary to the benefits of online technology in teaching, research has shown that the use of online teaching methods has been associated with teacher stress with negative impacts on teaching effectiveness (13).

In addition to the numerous factors mentioned above that can generate stress, it has also been argued that individual variables such as gender, can influence it. Although studies from different countries support this view, research on the relationship between stress and gender among university professors is still inconclusive. For example, while recent research with university professors in Spain (14), as well as Brazil (15) found higher stress scores in women compared to men, a survey by Fang et al. (2) with Chinese university professors found that job stress scores were significantly higher in men than in women. Lastly, no statistically significant differences were found among university professors in Bulgaria regarding gender (16).

In Brazil, political and economic changes over the last 25 years have led to significant transformations in the university system, particularly within public universities (17). These changes include an increase in enrollment numbers, larger class sizes, and the creation of evening and distance learning courses, all without a proportional adjustment in infrastructure or the number of required professors (18). Given these changes, and the above-mentioned literature, it is likely that university professors in Brazil, especially those in the public universities, are experiencing intensified workloads and deteriorating work conditions, placing them at high risk of occupational stress and its consequences (19). Despite these findings, research on occupational stress among Brazilian university professors remains in its early stages.

Based on these considerations, this research focuses in two main objectives: Firstly, to evaluate the incidence and the forgotten effects (20), sources, and symptoms of occupational stress in a sample of professors from a Brazilian public university. Secondly, to analyze the effect of gender on the relationship between the sources of stress and its symptoms.

## 2 Materials and methods

### 2.1 Participants

The studied population was composed of 154 university professors from a Brazilian Federal University Medical School. Finally, 81 volunteers participated in the research. The participation rate was 52.6%.

Regarding the demographic profile, 56% of the participants were female. The predominant age range was 30–39 years (56%). Most professors were married and live with a spouse (80.2%). The majority (72.8%) had a doctorate degree and have been working as a professor for more than 6 years (71.6%).

### 2.2 Procedure

Once the review board of the University approved the study, a letter was sent to the Human Resource Department, seeking collaboration to conduct the research. All professors working in the School of Health Studies of the university were mapped.

The questionnaire link was forwarded to the e-mail addresses of all permanent professors at the medical school. It was distributed electronically accompanied by a letter indicating the research objectives and the researchers' name, and a free and informed consent form. Faculty members were required to review and agree to the terms before proceeding with the questionnaire. This ensured that participants were fully informed about the study's objectives, procedures, potential risks, and benefits.

By providing their consent, participants confirmed their voluntary participation and the confidentiality of their responses. This step was essential to uphold ethical standards and comply with legal requirements for data collection and processing. Respondents were guaranteed the anonymity of individual responses, ensuring that individual subjects could not be identified, as indicated in the informed consent. The personal data collected was only used for group data analysis, further protecting the privacy of the participants.

### 2.3 Instruments

The research instrument used was a questionnaire adapted and validated in Portuguese by Zille (21), which was structured in two parts. The first part addressed demographic and occupational data (gender, age, educational level, seniority), lifestyle (smoking, drinking alcohol, physical activity), and health problems of the respondent.

The second part collected data related to stress. It had 53 items structured in three constructs: sources of stress derived from work (SSW) composed of 26 questions (i.e., *The students' indiscipline has burdened me a lot in the classroom*), sources of stress derived from the individual (SSI), with 7 questions (i.e., *How often did you take life in a very fast pace, doing more and more work in less time during the last 6 months?*), and stress symptoms (SS), with 20 questions (i.e., *How often did you feel pain in the muscles of the neck and shoulders during the last 6 months?*). The instrument used a Likert-type scale with five degrees of response for each statement (1-never, 2-rarely, 3-sometimes, 4-frequent, 5-very frequented). The composite reliability of the constructs was: SSW (0.88), SSI (0.71), and SS (0.90) (21).

### 2.4 Data analysis

To analyze stress levels, based on stress symptoms, the criteria established by Zille (21) was adopted, in which four levels of stress intensity were established with the following reference values on a 5-point scale: absence of stress: < 1.75; moderate stress: ≥1.75 and < 2.46; intense stress: ≥2.46 and < 3.16; very intense stress: ≥3.16.

Univariate statistics were used to analyze stress levels and symptoms, sources of stress resulting from the nature of the work and those arising from the individual. Spearman correlation was used to test the relationship between sources of stress and stress symptoms. The data collected were processed using the statistical software IBM SPSS Statistics version 25.

Moderation models were tested with PROCESS for SPSS version 4.3.1 (model 1) (22). The models tested the relationship between sources of stress derived from work (SSW) and stress symptoms (SS), and sources of stress derived from the individual (SSI) and stress symptoms, both moderated by gender.

Finally, the forgotten effects theory (20) was applied to understand the incidence relationships between source of stress (cause) and symptoms of stress (effect), identifying direct causalities and those initially overlooked but having significant impact, or “forgotten effects.” This algorithm has been calculated with the application available in the Royal Academy of Economic and Financial Sciences–Barcelona Humanist Economy (23).

## 3 Results

The data revealed that, with regard to lifestyle and health status, only 2.5% of respondents reported regular smoking. The majority (74.1%) consumed alcoholic beverages and 86.7% of this group stated to consume 1–5 units of alcoholic beverages per week. The majority (63%) stated that they performed physical activities three to four times a week. Finally, 37% of the participants reported having some health problem such as hypertension (33.3%), gastritis (16.7%), depression (13.3%) and anxiety (10%), during the 6 months prior to this research.

Table 1 shows that 70.4% of the surveyed professors had some level of stress and 24.7% had intense to very intense stress (17.3% had intense stress, and 7.4% had very intense stress).

**Table 1:** Descriptive analysis of the occupational stress indicator, stress symptoms, sources of stress derived from individual (SSI), and sources of stress derived from work (SSW).

	**Variables**	***n***	**%**	**Mean**	**SD**	**Min**	**Max**
Stress level	Absence of stress	21	25.9	1.51	0.15	1.15	1.75
	Moderate stress	40	49.4	2.12	0.19	1.85	2.45
	Intense stress	14	17.3	2.77	0.19	2.5	3.05
	Very intense stress	6	7.4	3.63	0.46	3.25	4.45
Stress symptoms (SS)		81	100	2.19	0.62	1.15	4.45
Sources of stress derived from individual (SSI)		81	100	3.41	0.80	1.57	5
Sources of stress derived from work (SSW)		81	100	2.24	0.62	1.08	4.5

Related to the stress sources, participants have medium-high levels on individual factors and low levels on work factors.

The symptoms of stress scoring above 3 (in a 1–5 scale) were fatigue (3.07) and nervousness (3.04). On the other hand, all the sources of stress at the individual level, except “Having to do work activities well above technical capacity and/or recent learning activities, which you still do not fully master” scored above 3. The highest scores were “Take life in a very fast pace, doing more and more work in less time” (3.78), “Having a busy day with a series of commitments, with little or no free time” (3.64) and “Not being able to disconnect from work activities” (3.62). For the work dimension, only 2 sources scored above 3, “Carrying out several activities at the same time, with a high degree of demand” (3.44) and doing “A complex job, and it leaves me worn out/very tired” (3.25).

### 3.1 Association between sources of stress and stress symptoms

As can be seen in Table 2, sources of stress derived from individual and from work had a high and significant correlation (0.66). Additionally, we correlated both sources of stress with the symptoms. Using cigarettes or alcohol to relieve tension had no relation with any of the sources of stress. Anxiety, chest pain, palpitations, stomach upset or pain in the stomach had no relation with the sources of stress derived from individual, while they had relation with sources of stress derived from work. The highest levels of relationship were found between fatigue (0.431) and irritability (0.444) with stress derived from individual, while for stress derived from work, anguish (0.594), depression (0.515), and pain in the muscles of the neck and shoulders (0.576) were added to the previous.

**Table 2:** Spearman correlations between sources of stress and stress symptoms.

	**Sources of stress derived from individual (SSI)**	**Sources of stress derived from work (SSW)**
Nervousness	0.361^**^	0.430^**^
Anxiety	0.243	0.462^**^
Anger	0.305^*^	0.366^**^
Anguish	0.288^*^	0.594^**^
Fatigue	0.431^**^	0.652^**^
Irritability	0.444^**^	0.590^**^
Depression	0.298^*^	0.515^**^
Tension headache	0.348^*^	0.425^**^
Insomnia	0.342^*^	0.325^*^
Pain in the muscles of the neck and shoulders	0.312^*^	0.576^**^
Discreet chest pain under tension	0.165	0.301^*^
Palpitations	0.221	0.357^*^
Stomach upset or pain in the stomach	0.268	0.319^*^
Lump in the throat	0.289^*^	0.423^**^
Vertigo	0.289^*^	0.404^**^
Lack or excess of appetite	0.382^**^	0.402^**^
Loss and/or fluctuation of sense of humor	0.350^*^	0.453^**^
Using cigarettes to relieve tension	−0.024	0.036
Use of alcoholic beverages to relieve tension	0.159	0.207
Panic	0.303^*^	0.393^**^
Sources of stress derived from individual		0.660^**^

^*^p < 0.01 and ^**^p < 0.001.

Finally, only gender was shown to moderate the relationship between work-derived stress sources and their symptoms (Table 3).

**Table 3:** Gender moderation effect in the sources of stress-stress symptoms relationship.

	**Model summaries**				**Coefficient**	**Sub-dimension specifics**			
	*R* ^2^	***F***	Δ*R*^2^	***F***Δ		***B***	***SE B***	***t***	***p***
SSI	0.3066	11.349^**^	0.0049	0.5387	b1	0.3087	0.1187	2.5996	0.0112
					b2	0.2871	0.1056	2.7197	0.0081
					b3	0.1085	0.1478	0.7340	0.4652
SSW	0.6076	39.740^**^	0.0238	4.663^*^	b1	0.1825	0.0916	1.9915	0.05
					b2	0.5177	0.1174	4.41	< 0.001
					b3	0.3265	0.1512	2.1595	0.0339

^*^p < 0.01 and ^**^p < 0.001.

SSI, Sources of stress derived from individual; SSW, Sources of stress derived from work.

**Figure 1:**
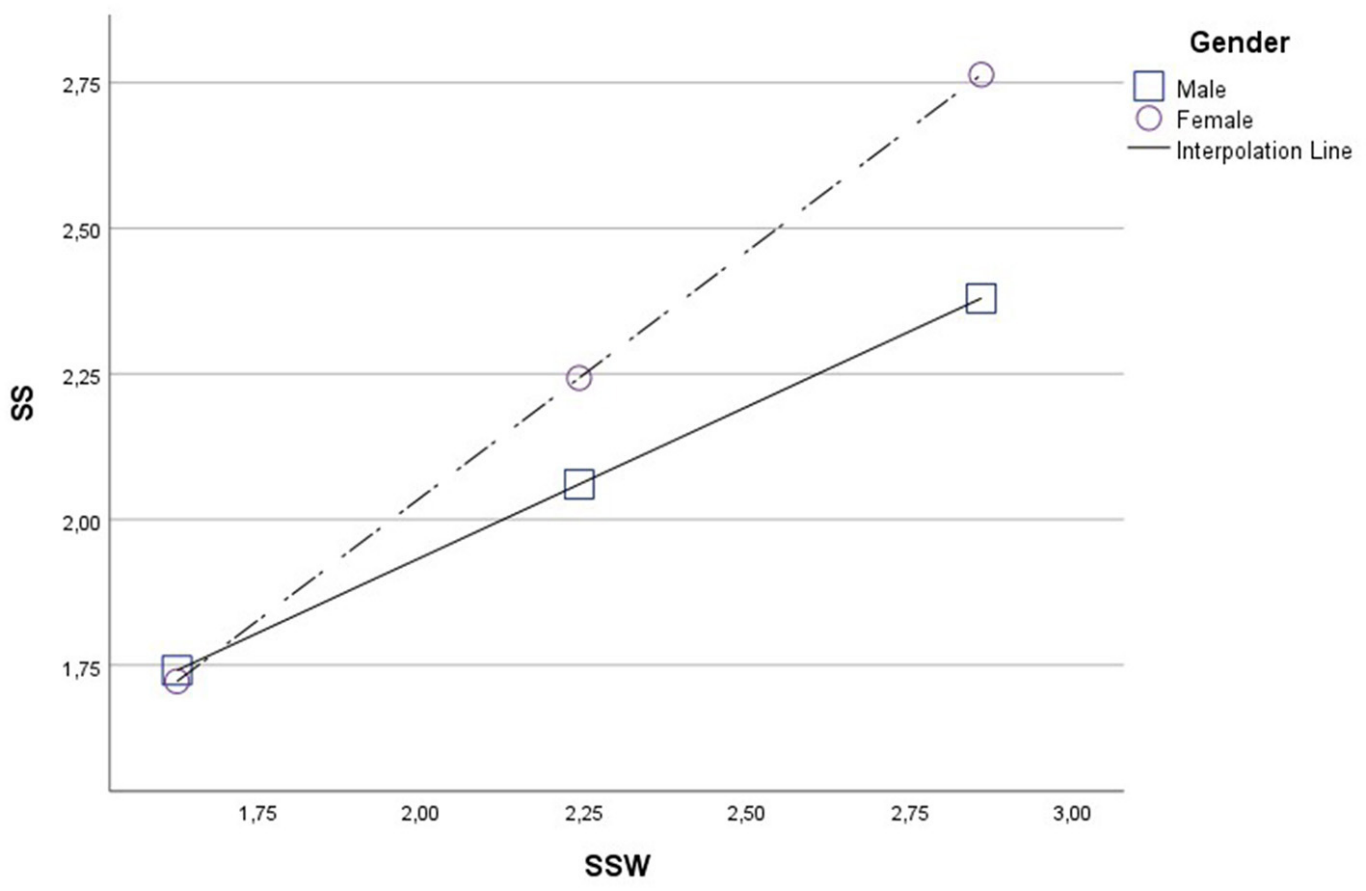
Gender moderation effect in the sources of stress derived from work (SSW)–stress symptoms (SS) relationship.

While the effect of work-related stressors is positive and significant for both men and women, in the case of women, the effect is more intense than in men as sources of stress derived from work increase (Figure 1).

### 3.2 Forgotten effects between stress sources and symptoms

The forgotten effects (20) were defined as the set of pairs of elements evaluated is the direct incidence matrix (M), which shows the cause-effect relationship by the corresponding set A (causes–sources of stress) and set B (effects-symptoms of stress), the matrix effects with itself (B), as well as cause with cause (A) (Table 4).

**Table 4:** Matrixes M, B and A.

		***b*1**	***b*_2_**	***b*_3_**	**…**	***b_*m*_***
*M* =	*a*1	μ*a*_1_*b*_1_	μ*a*_1_*b*_2_	*μa*1 *b*3	…	*μa*1 *bm*
	*a*_2_	μ*a*_2_*b*_1_	μ*a*_2_*b*_2_	*μa*2 *b*3	…	*μa*2 *bm*
	*a*_3_	μ*a*_3_*b*_1_	μ*a*_3_*b*_2_	*μa*3 *b*3	…	*μa*3 *bm*
	.	…	…	…	…	…
	*a_*n*_*	μ*a*_*n*_*b*_1_	μ*a*_*n*_*b*_2_	*μanb*3	…	*μanbm*
		***b*** **1**	*b*_2_	*b*_3_	***…***	**bk**
*B* =	*b*1	μ*b*_1_*b*_1_	μ*b*_1_*b*_2_	*μb*1 *b*3	…	*μb*1 *bk*
	*b*_2_	μ*b*_2_*b*_1_	μ*b*_2_*b*_2_	*μb*2 *b*3	…	*μb*2 *bk*
	*b*_3_	μ*b*_3_*b*_1_	μ*b*_3_*b*_2_	*μb*3 *b*3	…	*μb*2 *bk*
	*bm*	*μbmb*1	*μbmb*2	*μbmb*3	…	*μbmb*k
		***a*** **1**	*a*_2_	*a*_3_	***…***	***ak***
*A* =	*a*1	μ*a*_1_*a*_1_	μ*a*_1_*a*_2_	μ__*a*_1a3_	…	μ__*a*_1ak_
	*a*_2_	μ*a*_2_*a*_1_	μ*a*_2_*a*_2_	μ__*a*_2a3_	…	μ__*a*_2ak_
	*a*_3_	μ*a*_3_*a*_1_	μ*a*_3_*a*_2_	μ__*a*_3a3_	…	μ__*a*_2ak_
	.	…	…	…	…	…
	*a_*n*_*	μ*a*_*n*_*a*_1_	μ*a*_*n*_*a*_2_	μ__*ana*_3_	…	*μ_*anak*_*

The max-min convolution of the three matrices is performed [A]o[M]o[B] = [M^*^]. The new resulting matrix includes incidence relationships between the direct or indirect causes and effects. The difference is made between the matrix of direct incidences and the effects of second generation; in this sense, the relationships that have been forgotten in the decision process is [M^*^]–[M] = [D].

**Figure 2:**
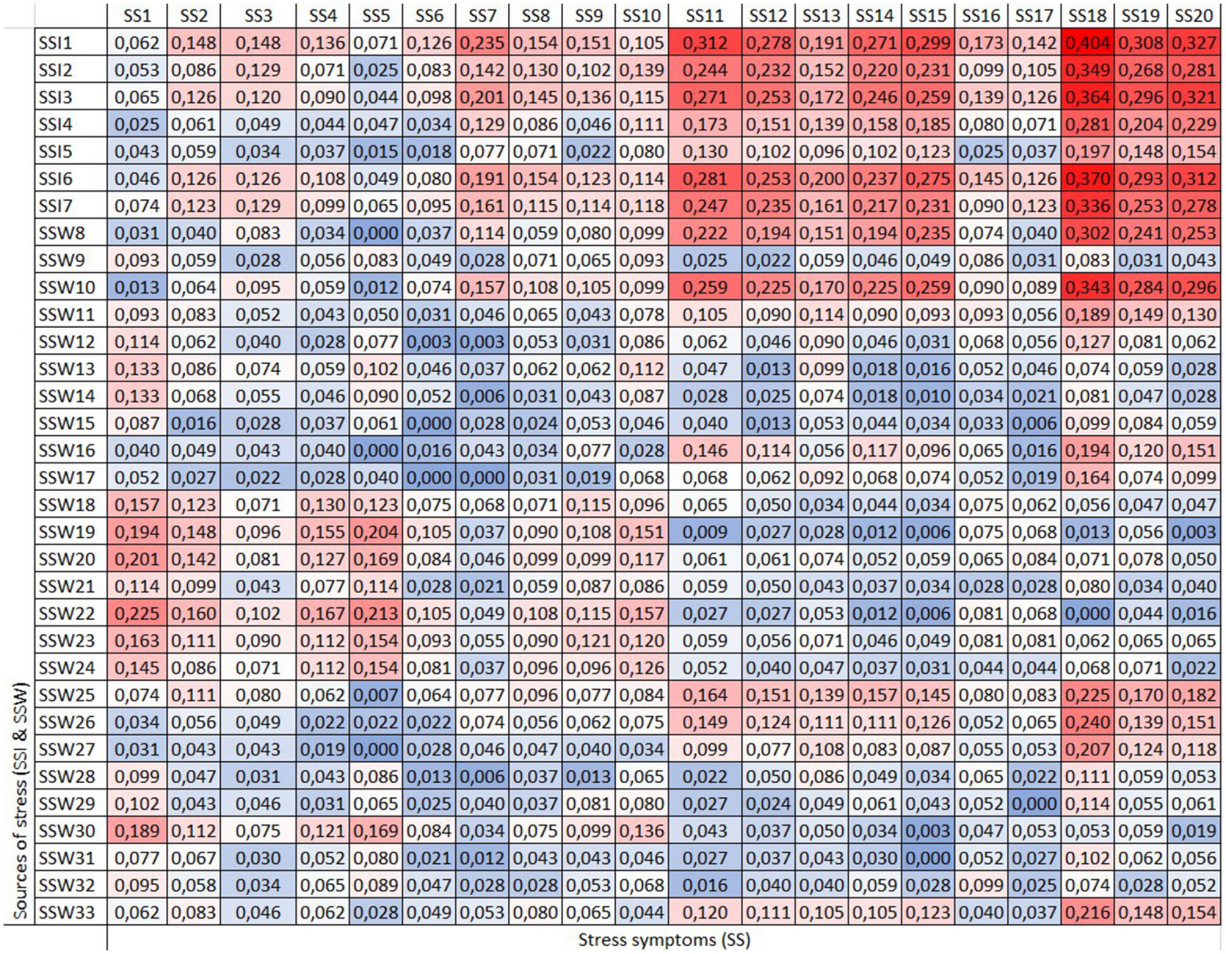
Matrix (D). Global sample.

The matrix analysis (Figure 2) reveals that discrete chest pain under tension (SS11), smoking cigarettes (SS18) or consuming alcoholic beverages (SS19) to relieve stress and experiencing panic symptoms (SS20) are associated with a forgotten effects score exceeding 0.3, especially related to “Take life in a very fast pace, doing more and more work in less time” (SSI1). Additionally, smoking is associated with forgotten effects related to “Often thinking and/or doing two or more things at the same time, with difficulty completing them” (SSI2). Finally, smoking and feelings of panic are associated not only with workload challenges but also with “Not being able to disconnect from work activities” (SSI3).

The use of cigarettes as a coping mechanism shows the highest forgotten effect related to “Taking life at a very fast pace, doing more and more work in less time” (SSI1) with a coefficient of 0.404. For this reason, we analyzed other factors indirectly influencing this relationship.

**Figure 3:**
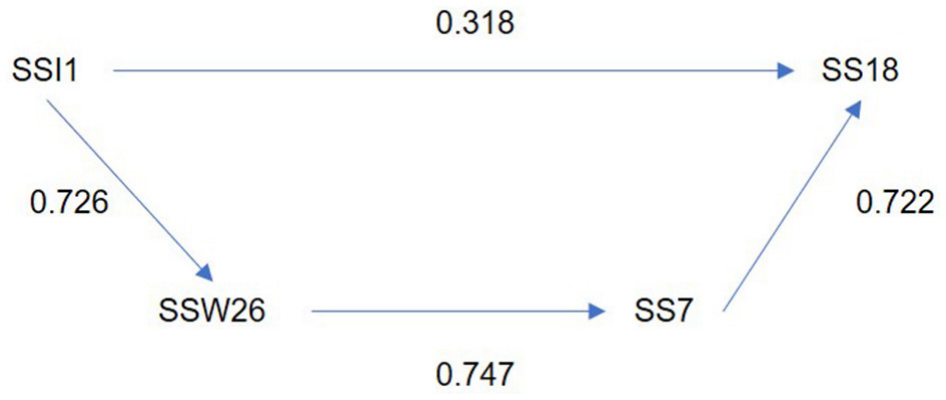
Direct and indirect effect between source of stress “take life in a very fast pace, doing more and more work in less time” and using cigarettes to relieve tension.

As can be seen in Figure 3, the symptom of using cigarettes to relieve tension shows a significant forgotten effect influenced by the factors “Taking life at a very fast pace, doing more and more work in less time” (SSI1) and “The excessive number of working hours” (SSW26). These factors, in turn, affect “Periods of depression (sadness, apathy, isolation, lack of energy)” (SS7), which also influences the symptom of using cigarettes to relieve tension (SS18).

### 3.3 Forgotten effects between stress sources and symptoms considering gender

**Figure 4:**
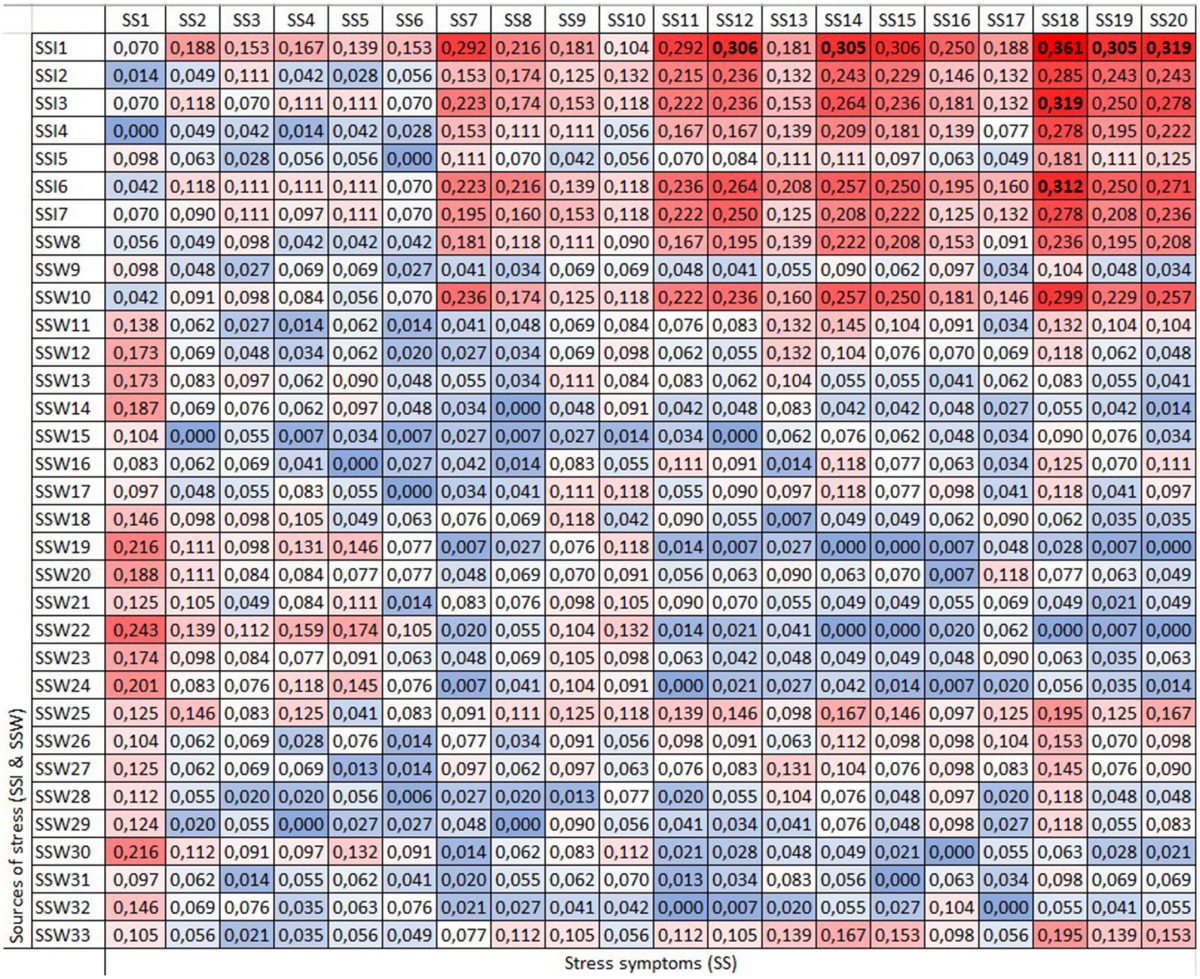
Matrix (D). Men's sample.

When the data were analyzed from a gender perspective, men exhibited stress symptoms with a forgotten effect exceeding 30% associated with three main individual sources of stress: “Taking life at a very fast pace, doing more and more work in less time” (SSI1), as well as “Not being able to disconnect from work activities” (SSI3) and “Having a busy day with a series of commitments, with little or no free time” (SSI6) (Figure 4).

The source of stress “Taking life at a very fast pace, doing more and more work in less time” (SSI1) is the one that shows the highest forgotten effects on stress symptoms, having an impact above 0.30 on palpitations (SS12), lump in the throat (SS14), dizziness, vertigo (SS15), using cigarettes and alcohol to relieve tension (SS18 and SS19), and panic (SS20).

Additionally, “not being able to disconnect from work activities” (SSI3) and “having a busy day with a series of commitments, with little or no free time” (SSI6) had higher forgotten effects on using cigarettes to relieve tension (SS18).

**Figure 5:**
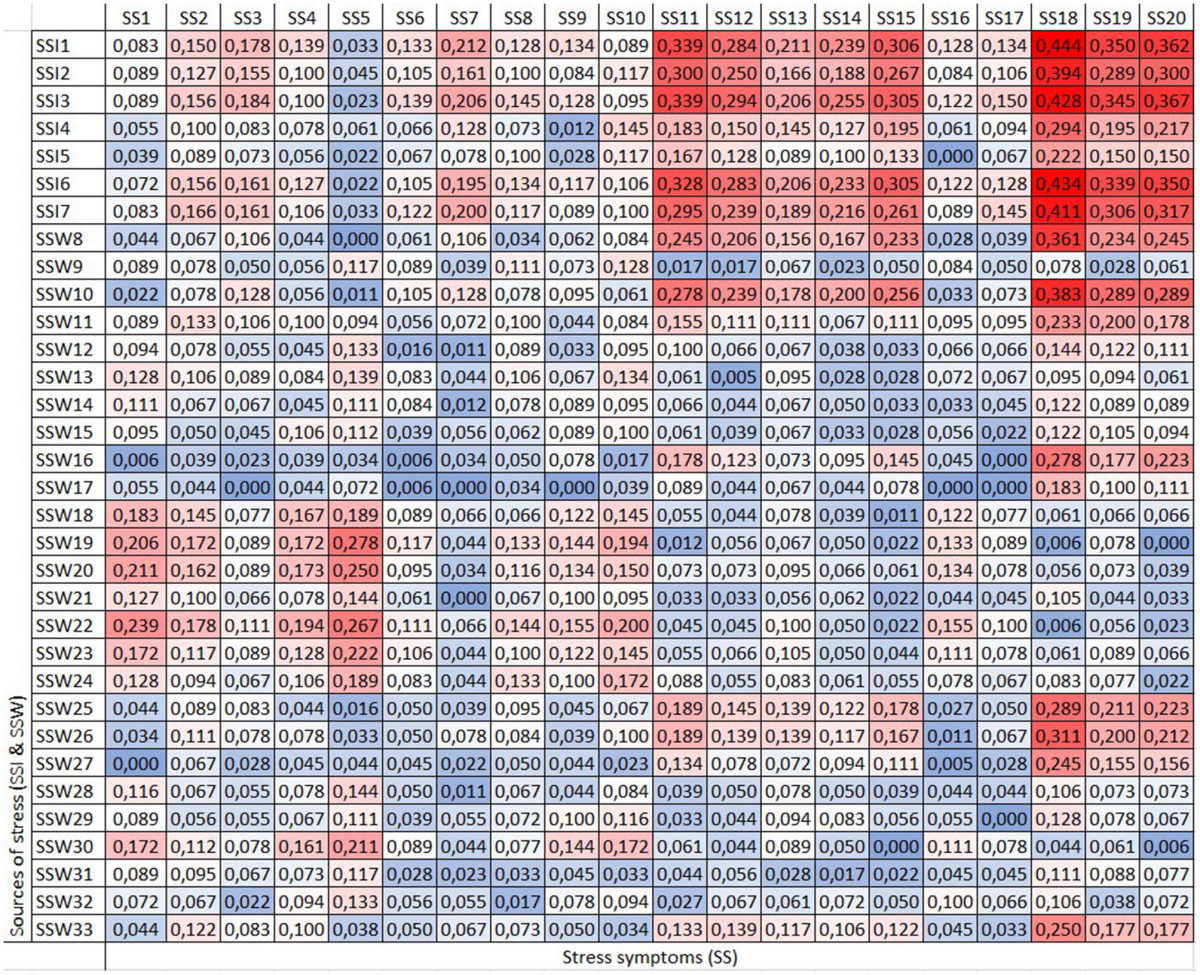
Matrix (D). Women's sample.

For women, most individual sources of stress presented forgotten effects over 0.30 in their relationship with discreet chest pain under tension (SS11), dizziness, vertigo (SS15), using cigarettes and alcohol to relieve tension (SS18 and SS19), and panic (SS20) (Figure 5).

Comparing both genders, the direct relationship among “Taking life at a very fast pace, doing more and more work in less time” (SSI1) and discrete chest pain under tension (SS11) was 0.417 for women; however, when considering the forgotten effects, the relationship increased by 0.339, resulting in a total adjusted relationship of 0.756. In contrast, men showed a more precise identification of this symptom, with a direct relationship of 0.465 with SSI1. For them, the forgotten effects were minor (0.292), and consequently the adjusted relationship was 0.757, similar to the value for women.

The symptom that shows the highest forgotten effects, for both men and women, is the use of cigarettes to relieve stress. Nevertheless, women exhibit a greater forgotten effect (0.444) compared to men (0.361). For men, only three sources of stress showed a forgotten effect >0.3. These sources include stress related to “Take life in a very fast pace, doing more and more work in less time” (SSI1) (0.361), “Not being able to disconnect from work activities” (SSI3) (0.319), and “having a busy day with a series of commitments, with little or no free time (SSI6) (0.312).

**Figure 6:**
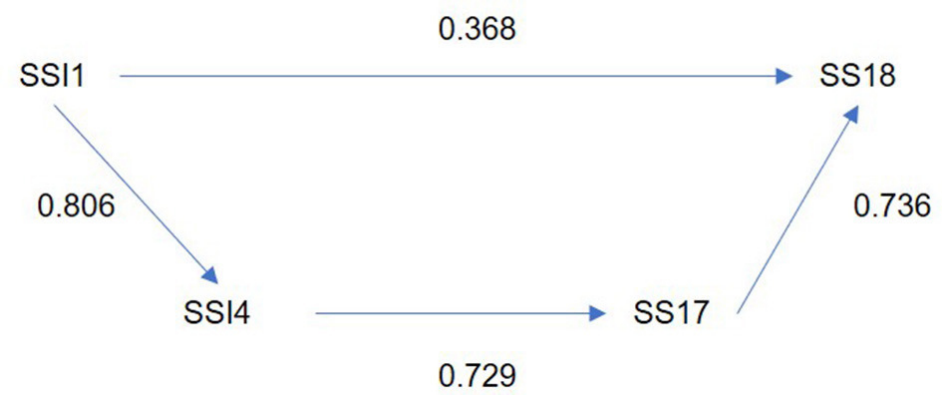
Direct and indirect effect between source of stress “take life in a very fast pace, doing more and more work in less time” and using cigarettes to relieve tension (Men).

Figure 6 shows the direct and indirect effects between “Take life in a very fast pace, doing more and more work in less time” (SSI1) and the use of cigarettes to relieve tension (0.729–0.368 = 0.361) for men. This relationship is mediated by “assuming, in the context of work, very challenging commitments, beyond limits” (SSI4) with experiencing a loss or fluctuation in sense of humor (SS17).

However, for women, this level of omitted incidence (0.3) has been directly identified in certain individual and organizational sources of stress: “take life in a very fast pace, doing more and more work in less time” (SSI1), “Often thinking and/or doing two or more things at the same time, with difficulty completing them” (SSI2), “not being able to disconnect from work activities” (SSI3), “having a busy day with a series of commitments, with little or no free time” (SSI6), “having rest times (after hours, holidays and weekends) taken up by work” (SSI7), “I do a complex job, and it leaves me worn out/very tired” (SSW8), “the work I perform consists of carrying out several activities at the same time, with a high degree of demand” (SSW10) and “the excessive number of working hours is considered by me as a major source of tension and/or a feeling of wear and tear” (SSW26).

**Figure 7:**
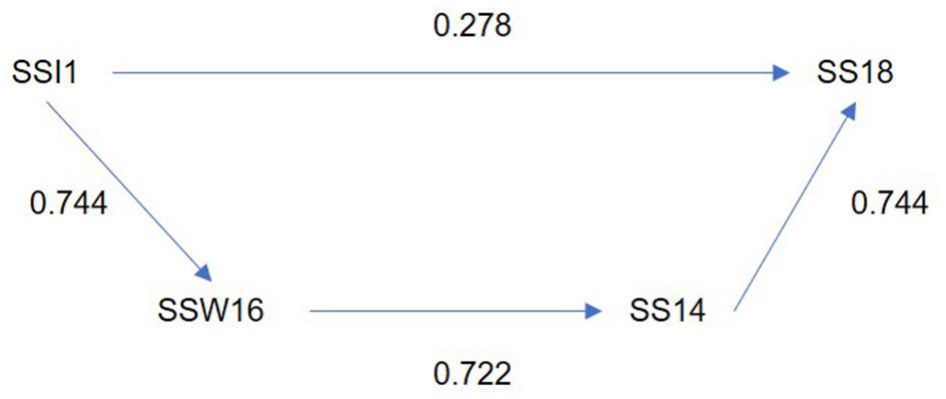
Direct and indirect effect between source of stress “take life in a very fast pace, doing more and more work in less time” and using cigarettes to relieve tension (Women).

Using the same source of stress, “take life in a very fast pace, doing more and more work in less time” (SSI1), and the stress symptom using cigarettes to relieve tension (SS18), we observe that the omitted effect follows another path through “living with “spreaders” (stressed, anxious, emotionally unbalanced individuals)” in the organizational context (SSW16) and lump in the throat (SS14) (Figure 7). The relationship between the stress symptom of using cigarettes to relieve tension and the source of stress shows a forgotten effect when comparing the direct relationship (0.278) to the indirect one (0.722), resulting in a difference of 0.444.

## 4 Discussion

The main objective of this research was to identify the sources and symptoms of occupational stress among Brazilian university professors and examine the impact of gender on this relationship.

The analysis of occupational stress among university professors in this study revealed that 70.4% experienced some level of stress. These findings align with previous research conducted in Brazil, which reported similar results (8, 19).

It is important to note that the majority of recent studies on occupational stress in Brazil have concentrated on healthcare professionals or primary and secondary school teachers (24). A significant gap persists in the literature concerning university professors, as recent studies addressing this group are descriptive and do not account for the mechanisms that explain the relationship between different types of stressors and physical and psychological responses. This highlights the need for more research to understand the unique stressors and challenges faced by university professors.

The primary physical and psychological symptoms of stress identified among the participants, such as neck and shoulders muscle pain and tension headache, specially during COVID-19 period (10), as well as common mental disorders as fatigue, nervousness, anxiety, or anguish (25), have also been noted in prior studies.

Additionally, this research analyzed the forgotten effects to uncover hidden relationships between sources of stress (causes) and stress symptoms (effects) among university professors. Through matrix analysis, we identified significant indirect associations where high workload and demands contribute to behaviors like smoking and alcohol use for stress relief, as well as symptoms such as discrete chest pain and feelings of panic. These stressors were also linked to difficulties in disconnecting from work and managing multiple commitments.

The meta-analysis by Heikkilä (26) already indicated that smoking and drinking habits were associated with work stress, though not causally. A recent Canadian study, encompassing over 35,000 participants, revealed that average alcohol consumption was significantly higher among individuals with poorer mental health perceptions and elevated work stress levels. Furthermore, alcohol consumption showed a positive association with smoking behaviors (27). Consistent with these findings, recent research among university professors in Brazil also identified alcohol as the substance most commonly associated with work-related stress (28).

The present research goes further by revealing how stressors such as workload demands contribute to behavioral responses and psychological symptoms. This deeper understanding enhances our grasp of workplace stress dynamics, suggesting targeted interventions that address both direct stressors and underlying contextual factors to effectively promote the wellbeing of academic professionals. In this sense, our findings are consistent with those of Amstrong et al. (29), who demonstrated that organizational stress in firefighters indirectly influences outcomes through mediating factors, highlighting the broader impact of workplace stress on individuals' wellbeing and performance.

Gender was found to moderate the relationship between work-derived stress sources and their associated symptoms. The effect of work-related stress on women was more intense compared to men as the sources of stress increased. Previous studies (30) have indicated that while both male and female teachers (specifically high school teachers) perceived similar levels of overload and emotional exhaustion, female teachers were more likely to adopt inadequate coping strategies, experiencing more severe and pronounced symptoms of stress.

The differential use of cigarettes for stress relief between men and women can be explained by two distinct substance use pathways. Men are more likely to follow the escapist path, using cigarettes to avoid or escape stress. In contrast, women tend to follow the presenteeism path, using cigarettes to maintain productivity and presence despite experiencing stress (31).

Finally, our study reveals a significant forgotten effect associated with “Living with ‘spreaders' (stressed, anxious, emotionally unbalanced individuals) in the organizational context.” This finding suggests that the social and emotional context of the personal environment can significantly influence perceived stress levels among female teachers, as previously noted by Yeom and Lee (32).

These insights highlight the critical need for developing targeted interventions that address the specific coping mechanisms and stress responses of female teachers to mitigate their risk of heightened stress levels.

By employing a framework that identifies forgotten effects, this research enhances understanding of how specific stressors indirectly contribute to stress symptoms, beyond direct causal links. Such insights are pivotal for developing targeted interventions that address both overt stressors and underlying, less obvious contributors to workplace stress. In this sense, the present research contributes to the consolidation of available knowledge about occupational stress in university professors in Brazil, including gender as a moderator.

The main limitation of the present study is the fact that the sample, despite being statistically representative, is small and was taken from a single public university in Brazil. Thus, future research should advance on this topic by involving professors from public and private universities, at both national and international levels, to map the propensity to occupational stress and its consequences in this professional category globally, thereby consolidating the literature on the subject.

## 5 Conclusion

The findings of the present research have significant practical implications for university administrators and policymakers. Implementing interventions to address both individual and work-related stressors (high workloads and organizational demands) can help mitigate the negative impact of stress (both physical and psychological) on university professors.

Additionally, indirect effects, such as smoking and alcohol use as coping mechanisms, were unveiled, shedding light on the broader behavioral and psychological consequences of workplace stress.

Finally, given the results obtained regarding gender differences, gender-sensitive strategies should be employed to support female faculty members, who may be more vulnerable to the effects of work-related stress. By effectively recognizing and managing occupational stress, universities can foster a healthier and more supportive work environment for their academic staff, ultimately benefiting both faculty and students.

## Data Availability

The raw data supporting the conclusions of this article will be made available by the authors, without undue reservation.
